# “It's like you are just a spectator in this thing”: Experiencing social life the ‘aspie’ way

**DOI:** 10.1016/j.emospa.2009.02.001

**Published:** 2008-12

**Authors:** Sara Ryan, Ulla Räisänen

**Affiliations:** DIPEx Health Experiences Research Unit, Department of Primary Health Care, University of Oxford, Old Road Campus, Headington, Oxford OX3 7RF, UK

**Keywords:** Asperger syndrome autism, Ethnomethodology, Symbolic interactionism, Social encounters, Socialisation

## Abstract

This study explores the experiences of people with Asperger syndrome (AS) from a sociological perspective using the theoretical approaches of ethnomethodology and symbolic interactionism. In-depth interviews were conducted with 16 people with AS and three key themes of feeling different, trying to fit in and safe spaces are considered here. We suggest that people with AS develop a different symbolic capacity to most people and have difficulties in making sense of social encounters. While these difficulties can be overcome, to some degree, by developing strategies to try to fit in, this learning remains at a superficial level and is not internalised through the process of socialisation. Without being able to derive a firm sense of reality from spontaneous involvement in social encounters, participants feel “unruled, unreal and anomic” (Goffman, 1967: 135) and experience intense autistic emotion (Davidson, 2007a,b).

People diagnosed with Asperger syndrome (AS) are commonly characterised as having difficulties with social interaction, social imagination and communication (Attwood, 2006). These differences have been the focus of a substantial body of work often located within the disciplinary fields of psychology, neurology and psychiatry. In this paper, we think about these difficulties from a sociological perspective to explore what insights the work of interactionists and ethnomethodologists, such as Goffman, Blumer and Schutz can provide about experiencing space the ‘aspie’^1^ way. Interactionism involves a focus upon the processes of action and interaction (Blumer, 1969) while ethnomethodology offers an alternative way of understanding how people manage the complexity, uncertainty and density of social life by focusing on the ‘methods’^2^ people use to negotiate social life. We will start with a brief overview of sociological perspectives focusing on managing social encounters and an overview of the issues associated with AS. We then present an analysis of data from a qualitative study involving in-depth interviews with 16 people with AS. We suggest that examining the experiences of people with AS using these sociological insights and conceptual tools provides us with a window into aspects of social life more typically taken for granted. Three related themes emerging from participant interviews are discussed: not belonging, trying to fit in and the need for safe spaces. In conclusion, we argue that the different symbolic capacity of people with AS should be understood in terms of difference rather than deficit.

## 1 Maintaining social order

Contemporary theorists such as Bauman (2000) and Beck and Beck-Gernsheim (2002) argue that contemporary society is characterised by the fragility and fluidity of social bonds. Individualisation and the (arguably) accompanying weakening of a collective community sentiment (Puttnam, 2000) foster an enhanced sense of fear and anxiety about strangers, difference and otherness (Bauman, 1991; Hall, 1993). Because the capacity for danger, risk and uncertainty in everyday life is so potentially enormous we operate in ways that maintain an illusion of safety and security and this revolves around trust (Giddens, 1991) and reciprocity (Rawls and David, 2006). For Misztal (2001), social trust is based upon perceptions of normality which are, in turn, maintained by rule-following behaviour that makes social life predictable, reliable and legible. Familiarity and predictability are, therefore, commonly desired by people who want to engage in mutually understandable interactions (Sibley, 1995: 115). Misztal (2001), like Cahill (1987) and following Elias (1978), focuses on the importance of civility and highlights the way it smoothes and simplifies interactions, promoting greater trust and integration.^3^ For Cahill, people are emotionally committed to a;

code of ceremonial conduct which characterises our religion of civility thereby becomes the individuals' own inner moral code and colours his or her sense of the reasonable, the practical, the humane and the moral. (1987: 312)

This code is ceremonial, that is governed by etiquette, rather than substantive, comprising law, morality or ethics (Goffman, 1967: 55). And, for Cahill, failure to fulfil ceremonial expectations causes feelings of anger, embarrassment, revulsion or shame on the part of the person who fails to fulfil these obligations.

That the shared framework of reality is simultaneously sturdy and fragile is discussed by Giddens (1991), who suggests that our sense of ontological security rests on a sense of confidence in the reliability of people, which is acquired through socialisation. For people with AS, ontological security is, as will be demonstrated further on, elusive. There is an interesting dichotomy within the literature; on the one hand, the ceremonial rules regulating social encounters are held to be so taken for granted and ingrained that they are taken to be ‘natural’ as noted by Cresswell (1996), on the other hand, people have to maintain a constant vigilance and wariness in everyday life (Giddens, 1991; Goffman, 1969).

Goffman (1963, 1969) undertakes the work of illustrating the complex, ceremonial rules operating in interactions. For Goffman, people have a mutual obligation to facilitate social encounters by maintaining a “working consensus” (Goffman, 1969) and “fitting in” (Goffman, 1963). This entails individuals not only maintaining their own proper involvement in interactions but also ensuring that others present maintain theirs. Goffman (1967: 116) highlights the significance of this by describing the acting out of this obligation as “the spark that lights up the world”. Moral obligations, therefore, include being sympathetically aware of the kinds of ways in which others present can become spontaneously and properly involved, and then attempting to modulate one's own expression of attitudes, feelings and opinions according to the company (Goffman, 1967: 116). One of the underlying questions we are concerned within this paper is the moral obligation of neuro-typical people to facilitate and support the inclusion of people with AS in mainstream life.

Of course, there are constraints on the extent to which people will sympathetically support other peoples' participation in social encounters and Goffman (1963) offers us the principle of ‘civil inattention’ to cover this discrepancy. He suggests that people tend to only glance at others in a casual setting unless a person behaves out of turn, in which case stares and glares are effective mechanisms of social control.

Goffman (1967: 49) terms those who fall short of acting out their involvement obligation “faulty interactants” who make themselves and others uneasy in encounters. This pejorative term might be seen to place the blame with the individual rather than focusing attention on the rigidity of social expectations and normative behaviour. This draws parallels with medical versus social models of disability (see, for example, Oliver, 1996).

For this paper, the most important point in Goffman's analysis is his argument that;

All encounters represent occasions when the individual can become spontaneously involved in the proceedings and derive from this a firm sense of reality. (Goffman, 1967: 135)

Not being able to derive a firm sense of reality, a sense underwriting any hope of ontological security, leaves people feeling “unruled, unreal and anomic” (Goffman, 1967: 135). In some respects, Goffman's analysis of the order of interaction is Durkheimian, or: draws from Durkheim (1984) as it presents what could be described as a functionalist view of people working to maintain public order.

Emotions are clearly integral to this work, as anxiety, discomfort, embarrassment, shame, fear and so on may be related to the experience of disrupted encounters. As Davidson and Bondi (2004) suggest, emotions mediate the way the world is for us. Our sense of who and what we are is constantly (re) shaped by how we feel. By the same token, what we feel, or more importantly, how we learn to interpret what we feel is a product of existing in that world; it is a dialogical relationship. The emotional fallout of being considered a ‘faulty interactant’, combined with experiencing the accompanying feeling of anomie or detachment from mainstream life, can have a significant impact on people.

## 2 Asperger syndrome

The autism spectrum incorporates a range of disorders characterised by difficulties in social interaction, communication and social imagination/theory of mind,^4^ including Asperger syndrome (AS). Some people diagnosed with AS can be described as high functioning in some areas while struggling in others (Attwood, 2006). Difficulties in social interaction and communication can be attributed to several factors, not least the lack of mainstream understanding there is about the syndrome (Portway and Johnson, 2005). For people with AS, a difficulty in understanding body and facial language, sensory sensitivities such as hyper- or hyposensitivity of hearing, sight, smell, touch and/or taste and a tendency to interpret things very literally all contribute to making social life largely illegible and, as a result, unpredictable (Davidson, 2008). Given the significance of trust and predictability in social relations, it is perhaps not surprising that intense and unpleasant ‘autistic emotion’ (Davidson, 2007a) has been linked to the experience of AS.

There is considerable controversy surrounding the definition of AS (see, for example, Mayes et al., 2001; McLaughlin-Cheng, 1998) and the existing literature tends to present a stereotypical picture of people with AS as unemotional, non-communicative and anti-social (Stazmari, 2004). Recent non-clinical research as well as personal accounts written by individuals with AS do, however, present a very different picture, conveying the range and diversity of people with AS while demonstrating some striking similarities (see, for example, Davidson, 2007a; Grandin, 1996; O'Neill, 1999; Williams, 1995). The picture of the ‘aspie’ experience gained from this literature suggests alienation and a powerful sense of being able to view mainstream life without being able to access it fully. As Davidson (2007a: 669) writes;

These emotional states can form a long term background to the pronounced terrors, frustrations, embarrassments and revulsions that characterise daily attempts to engage with that other world.

Feeling alienated and not fitting in, of course, contributes to difficulties in maintaining a working consensus in social encounters, and people with AS could be identified by others as ‘faulty interactants’ in the sense Goffman wrote about. This concept, however, does not allow an engagement with the ‘aspie’ experience or perspective and, as this paper will demonstrate, there are places in which people with AS do fit in, underlining the importance of an engagement with the emotio-spatial hermeneutic (Davidson and Bondi, 2004).

## 3 Emergence and improvisation

For the reasons outlined above, we have taken an interactionist approach in this analysis, focusing on the meaning people with AS ascribe to their actions and their interpretations of the actions of others. An integral part of this approach is Mead's (1934) analysis of the development of the self. For Mead, one of the key stages to becoming a fully participating member of society is the development of the generalized other which involves being able to see things from the viewpoint of other people through the development of ‘symbolic capacity’.^5^

Building upon Mead's analysis, Blumer (1969) uses the term ‘emergent action’ to describe the course of action which emerges as a situation is defined and redefined by people. This calls for improvisation which, again, reinforces the idea of the complexity of encounters and the nuanced exchanges – both verbal and non-verbal – that occur when people interact. For interactionists, most people do not simply learn social rules and norms but internalise them, thereby allowing space for improvisation, adjustment and adaptation. People with AS may lack the socially appropriate skills to facilitate face to face interaction and so may not internalise the social rules and norms allowing them to improvise, adjust and adapt. Their experiences can, therefore, reveal different aspects of social life that would not otherwise be apparent.

The focus of ethnomethodology on the methods people use to negotiate social life is also relevant to our analysis. Schutz (1962) suggests that people draw upon common sense understandings of social actions in the natural attitude^6^ because social life would be too overwhelming to question or analyse every ‘trivial’ aspect of everyday life. This common sense attitude is a set of taken for granted ‘things’ among any given set of people who do not need comment or explanation (Schutz, 1962). While much research into the experiences of AS has been psychological, a sociological focus suggests that people on the autism spectrum may not develop this ‘common sense attitude’; they do not have access to a stock of knowledge which simplifies and smoothes individuals' journeys through the social world.

## 4 Methods

The data upon which this paper is based were part of a larger study exploring the everyday lives of people on the autism spectrum and parents of children on the spectrum in the UK. In-depth qualitative methods were used to understand how participants made sense of their lives, what sort of issues or life events mattered to them and how they negotiated the social world. This enabled us to tap into the knowledge and experiences of people's engagement with places and spaces; their everyday geographical ‘lifeworlds’ (Dyck, 1995). While the overall study included over 70 parents and people on the autism spectrum, the analysis here focuses upon the experiences of the 16 people in the sample who were diagnosed with AS and, in four cases, their neuro-typical partners.

The sample was recruited through a combination of support groups, online communities, advertising on national charity websites and ‘snowball’, or chain referral, sampling. A maximum variation approach (Sandelowski, 1995) was taken to incorporate participants of different age, gender, ethnicity, social class, geographical location, marital status, age at diagnosis and living arrangements (such as independent, independent supported living, with partner, and with parents). The sample comprised of people with varied backgrounds including students, volunteer workers, a retired scientist, a computer programmer, a kitchen worker, a classroom assistant, and people who were long-term unemployed. Five participants were married or in long-term relationships, two were divorced and the remainder single. Five participants had children and two had children also on the spectrum.

The research, funded by the Wellcome Trust, was part of the DIPEx Health Experiences Research project which aims to collect personal experiences of different health experiences to form sections on a website, www.healthtalkonline.org. These sections provide a resource for people experiencing similar health conditions and a teaching tool for health, medical, education and social care students and professionals. Interviews are filmed or audio recorded with participants' permission and video, audio or text extracts from the interviews are used on the website.^7^ Interviews generally are in two parts: first, people are asked an open-ended question; ‘Can you tell me about your experiences?’ This question commonly prompts chronological narratives which can last between 20 minutes and up to several hours. The second part of the interviews is based on a semi-structured interview guide, plus the probing of various aspects raised in the first part of the interview.

The interviews with people with AS involved adjusting this approach. When the information was sent out advertising the project in different places electronically, some people got in touch by email and said that they wanted to take part but had difficulty interacting socially face to face. They described finding it difficult to find words and explained that people often filled this gap for them, effectively preventing them from talking about their experiences. SR, who conducted the interviews, reassured participants that they would be able to take as much time as they needed without interruption and the interviews would be arranged at times and places that suited them to help them feel as comfortable as possible. The interviews largely took place at participants' houses with a few held in meeting rooms in hotels and support centres. One participant (Trisha) originally emailed to take part in the study but in further email contact said she felt she had nothing of value to contribute because she found it hard to find words in a conversation; she felt she was never listened to. She was offered the opportunity to tell her experiences electronically via an email interview and agreed to this format. The remainder agreed to a face to face interview.

Participants were largely resistant to the general question of ‘tell me about your experiences’ and requested a question–answer format during the interview. This was not surprising given the desire for people with AS to have clear, concise instructions. A semi-structured interview schedule was used flexibly allowing participants to take direction of the interview as much as they wanted to. The total length of the interviews was between 20 minutes and 3 hours, the average lasting around 1.5 hours. Four participants were interviewed with their partners.

The interviews were recorded and transcribed in full (including the length of pauses) and data management software was used as an organizational aid for thematic analysis. Data were open coded and categories and sub-categories were developed, repeatedly reviewed, compared and integrated using a constant comparative method (Morgan, 1993; Seale, 1999). Several themes emerged from the dialogue between the data, the existing literature and our knowledge of existing and relevant themes and topics within this area. Three related themes are discussed here: not belonging, trying to fit in and the need for safe spaces.

## 5 A different logic

All participants described a constant feeling of not belonging. This was framed both temporally and spatially. Participants recounted experiences from childhood and talked about how their interests differed from those of their peers; they enjoyed more individual, structured play and found team games, like football, or imaginary games, like ‘cops and robbers’, problematic because the rules were unclear or interpreted flexibly by other children. Imaginary play was difficult to comprehend;

It was when everyone was playing like cowboys and Indians and cops and robbers and things and I didn't get it because all these people were pretending to be like a cowboy or a robber and I didn't understand why. For obvious reasons, because they weren't, they were just a bunch of school kids running around. (Tom, aged 19)

This extract illustrates how participants question the underlying logic and rationale used by most people to make sense of ordinary events and behaviour. They apply a different logic to the social world, seeing and interpreting the social very differently. Following Mead's (1934) analysis discussed earlier, there is some gap here in developing a ‘generalized other’. Participants appeared to develop a different symbolic capacity, one in which they found it difficult to see how other people look upon the world (see, for example, O'Neill, 1999). Within psychological theorizing about autism, this has been termed the theory of mind (see, for example, Baron-Cohen et al., 1985) and has been theorized as a deficit rather than difference. We argue that new understandings can emerge from sociological engagement with this issue.

Study participants were very aware of this different symbolic capacity. Participants described how earlier difficulties with imaginary play and joining in with their peers were followed by difficulties in understanding the rules (or point) of everyday encounters and social greetings. One participant, for example, described how he had never understood the ‘inane-ness’ of much interaction, such as asking people how they were because the accepted answer was always ‘fine’ rather than a realistic description of how they were. Some people experienced a sense of not belonging even within their immediate family;

I never sort of really felt as though I belonged and I think that was highlighted particularly in my family, because, you know, I was so sort of very different from my parents and my sister was so very similar to my parents. It, it just sort of highlighted and made me almost feel as though basically sort of like a freak. You know you really had nothing in common with these people and with people in general and it was a very lonely feeling […] You know, loneliness, I think, sort of becomes the default setting. (Robert, aged 27)

Growing up feeling so distant socially and emotionally from their peers and family members lead some people to feel bad about themselves. Most of the participants described experiencing depression and several had thought about or attempted suicide.

Most participants felt that a barrier prevented them from being part of social life and while they could see what was happening, they could not join in. This finding is supported by the autobiographical literature (for example, Miller, 2003; Williams, 1995) and could, again, link back to the development of a different symbolic capacity; one that is incommensurate with mainstream socialising.

I remember just always sort of feeling as though I am not really the same as these people. You know, I don't really get why they do it. You know, it makes no sense to me. And it was always very much… I mean I have heard the analogy before as sort of viewing the world as through sort of a pane of glass. You can see it… (Robert, aged 27)

It is as if I am walking around, and everybody has got it, but me, because I have got Asperger's. You can say it sounds a bit silly, but it is absolutely true. And I have found this, with you know… it is as if we don't see something. We don't grasp something from an event. Something important. (Peter, aged 62)

Some linked this barrier to emotions; they described not ‘getting’ the emotional bond or connection that other people seemed to develop. A common perception of people with autism is that they appear emotionless but while participants talked about feeling emotionally detached from other people, crucially, this did not mean they did not experience emotions. One man, John, described himself as “a terminator,^8^ yes… a terminator but with feelings” while another participant said;

I just feel like a really tiny person in a huge big world and that sometimes it feels that it is just happening all over there somewhere and I am living in a bubble or living on the other side of a plate glass window to everybody else. It is like you are just a spectator in this thing, you know, and it is kind of like really hard being alive sometimes and I go through when I wonder just how much long I have got left, you know, because I really don't want all this pain in my life, living with pain. Daily. And it, it gets tiring and I don't want to keep hurting and I don't want to hurt every day and I don't want to struggle through things every day… and life just hurts, just hurts being alive. (Mary, aged 43)

This extract is an example of intense autistic emotion (Davidson, 2007a) offering an insight into what it feels like not to belong. Most of the participants talked about experiencing depression and presented their depression and AS as connected and interlinked. Some people talked about knowing that emotional intimacy was an integral part of ‘normal’ life but said they found it too uncomfortable to cope with.

In many ways I sort of wanted to have a relationship because that is what really you are supposed to do. That is what normal people do but being in that situation was just something I felt so uncomfortable I didn't want to have that sort of level of intimacy, you know, I do not want to share every detailed aspect of my life… I mean sharing sort of, you know, entirely every aspect of your life with somebody. I am not sure I could ever be truly comfortable with that. You know, like sharing a room with somebody. My God that would drive me nuts, you know, it would not be happening. (Robert, aged 27)

Again, this extract highlights the tension between being aware of what people are ‘supposed to do’ but not being able to do that. Participants demonstrated a high level of awareness of the appropriate social code and the weight of its expectation. Robert talked about having casual sexual encounters and he had a few relationships in the past but had come to realise that he would not be able to share his life with someone *in the expected social form*.^9^ Those participants in long-term relationships (with neuro-typical people) talked about the difficulties within their relationships because of their emotional detachment from their partners. This is discussed further in Section 6.

Part of feeling that people with AS did not belong was wrapped up in the difficulties many described in interacting, communicating and understanding social rules. Most people described being very literal and this, again, caused misunderstandings, confusion, distress, agitation and anxiety. As one participant, Tim, commented; “People say a lot of things that they don't mean, and they don't mean a lot of things that they do say”. They indicated particular struggles in phatic communication (Malinowski, 1923), a form of communication which functions primarily for social, interactional and emotive purposes and not to exchange information or facts. For Malinowski, phatic communication functions as a form of social bonding necessary to maintaining (neuro-typically) fluid, fluent interactional dynamics.

Davidson (2003: 115) suggests most neuro-typical people have a tacit ‘feel for the game’ rather than following explicit rules. However, AS participants in this study were clearly relying on formulaic codes that were not always appropriate. This lead to the experience of what one participant, Paul, called “social suicide situations” which he described as;

Sort of awkward moments, [3 second pause] like you say something stupid, and then realise you have said something stupid, and say something even more stupid, and or say something or do something awkward and then sort of combination of doing something awkward and saying something awkward and trying to make it funny and then making it even more awkward, making yourself look like a complete and utter idiot and then going all sort of red in the face and then hiding for days. (Paul, aged 17)

Goffman's (1967: 119) concept of interaction–consciousness is relevant here as Paul is demonstrating being consciously concerned “to an improper degree with the way in which the interaction, qua interaction, is proceeding”. The example Goffman uses to illustrate this concept is that of a party host worrying about the overall proceedings rather than becoming spontaneously involved in the official topic of the conversation. The irony here is that participants were interaction–conscious as part of the process of *trying* to fit in and join in the conversation and we return to this in the following section. Goffman (1967) also talks about the importance of having “safe supplies” handy to provide enough content to keep the conversation going and some participants used this strategy. Here Paul talks about his use of safe supplies;

I would, you know, find, even if it wasn't particularly interesting; “Oh look, butter, do you like butter?” “Yes, butter.” And even it if it was just like a two minute conversation I would just pick something randomly in the room, just like, talk about the microwave for five minutes and then realise I had talked about microwaves for five minutes, you know, and then it would, I would stop thinking about things in the room and then try and pick up I guess personality traits, or what people were wearing, or I guess if I noticed any differences, like if some one had had a hair cut I would try, I guess, and make conversation about that. (Paul, aged 17)

Of course, this is an experience that is probably familiar to many neuro-typical people but the level of intensity and frequency is substantial for people with AS who are unable, or find it difficult to, internalise social norms and values.

One final point that emerged from this theme of feeling different was that people with AS have a perceptive understanding of neuro-typical people which is often not reciprocated. For example;

Yet people can't understand that filling in a form for this that or the other or not turning up to an exam because you just freak out. Yet people can't understand why didn't you just go? Why didn't you just do this? Or do that? It is completely incomprehensible to them that you can do lots of what they perceive to them is something, you know, difficult, you can't do these simple tiny little things and it makes you feel like a complete and total idiot because you know you are not stupid, you know how trivial and how small these things are, and you can't do them. (Robert, aged 27)

This suggests that the symbolic capacity should not be interpreted in terms of a deficit, more as a difference. The extract also highlights how what are commonly perceived to be small, mundane social norms can present as massively significant and difficult to overcome for people with AS. It is as though there are two, incommensurable universes where something mundane, small and taken for granted in mainstream life is an alien, challenging and uncomfortable act for people with AS. The problem is that for people with AS this tension represents the overwhelming majority of their interactions and experiences because they live in a neuro-typical world.

Participants suggest that typical others found it hard to understand how they felt or to make sense of their emotional detachment and yet participants felt enormous pressure to try to reduce such differences, make sense of the social world and try to fit in. This was validated by the four neuro-typical partners who described their frustrations and the ways in which they had tried to make sense of and understand their partners' behaviours, actions and emotions, attempts which were not always successful.

## 6 A desire to ‘fit in’

One of the most striking themes emerging from the data was participants' awareness of their difference and the efforts they made to overcome it. This did not involve a desire not to be ‘aspie’ but a desire to learn to fit into mainstream society. While some participants enjoyed spending time with other people with AS there was little sense of collectively celebrating their difference.

Trying to fit in had emotional and spatial dimensions. Some people learned by watching other people and developing ‘tricks and ploys’ to facilitate their participation in social life. A few participants talked about forcing themselves to endure social situations they knew they would dislike, such as going to gigs or staying in the halls of residence at university, because they wanted to learn to overcome these difficulties and ease social interactions. A couple of participants described reading books about body language or self-help skills to learn how to interact ‘normally’. This process again was about learning a surface level of fitting in; it was not internalised or lived but learnt. A married participant, for example, talked about trying to develop his emotional communication to improve his relationship with his wife;

The problem with emotional communication is in both directions or all directions. I can't read my wife's emotions, except on a very broad brush; happy, angry. I would have difficulty putting in the finesse of ways. And the same thing applies to my own. I don't communicate my own emotions usually because my emotions don't communicate themselves to my mind. I know I have got emotions but I don't explain them. (Richard, aged 58)

He returned to this theme later in the interview and the following extract includes his wife's comments;

Sue: Yes, I mean on a number of occasions we tried various forms of counselling which hadn't really had any effect at all because the general aim of most sort of relationship counselling is to try to get both sides to appreciate the other person's point of view and that is something that Richard just couldn't do. He couldn't put himself into my shoes and see anything from my point of view.

Richard: I remember, the counsellor was saying, “You should take more account of your wife's feelings.” But even then I think I knew the problem was that I didn't know what her feelings were and that was why I wasn't taking account of them, so it wasn't helping. They were telling me to do something that I knew I wanted to do but I couldn't. (Richard, aged 58 and Sue, aged 56)

Again this extract highlights the way in which the participant knew what the problem was but did not have the appropriate symbolic capacity to resolve it. He was not able to put himself in the shoes of his partner to have some idea of how she felt. The foundation of most therapy/counseling logic is the question “tell me how you feel” and this question is overwhelming and impossible to tackle for people with AS.

Furthermore, the inability to reach the stage of taken for grantedness meant that interactions remained conscious activity – ‘conscious work’ as Richard called it – a process that had limitations and was tiring, draining and constant.

You do learn strategies from an early age I think and the problem is with people probably on the spectrum is that you have got a lot of information that you need to store away because you have to remember the strategies for those situations [um] because it doesn't come naturally so you have to pull that out of your little film cabinet that you have got in your head and play it quite quickly so you know what to do. It is not inherent really. (Tim, aged 44)

One of the factors working against knowledge becoming common sense was the very aspects of social life that encourage the development of order and routine on everyday lives; the unpredictability of social interactions. A few participants, for example, talked about how difficult it was to get back to something when they had a break from it. In this extract, Caroline discusses her work as a volunteer gardener;

And it is also keeping at it as well though, because if I sort of take a break from where I volunteer, if I don't do it for a bit, then when I go back it feels like I am doing it all over again new. And I feel like the first time I went back after I had been on holiday for a week, I thought I hope no one talks to me, I really hope no one talks to me. I can't deal with it. (Caroline, aged 25)

This extract again underlines the learnt rather than lived way participants' experienced social life. Because social rules are not internalised or felt, they have to be constantly re-learnt over and over and this is a harder task if there is a gap between experiencing particular situations.

The way in which people did not ‘get it’ was effectively summarised by Michael using metaphor; he described how every morning was like the first 20 minutes of the film *Saving Private Ryan* – a film which begins with a chaotic, noisy, violent war scene – an analogy he had heard a speaker use at a conference. For him everything took twice as much concentration and twice as much energy. Another participant described;

I suppose it is like how did you feel when you went for your first interview ever? You know, if you can imagine that same interview like fifty times a day. And then you have got an idea of what it is like because … and then you get, you cover things up as well, because you do want to fit in and you do want to be like everybody else. So you, even if you don't get things, you make it look like you do get things and then you are thinking of what it was that was said, because you want to find out ways so you carry that around in your head all day as well, as well as everything else. And yes, and then you get your overload. (Tim, aged 44)

The use of metaphors and analogies suggests shared meanings and understandings and this is, perhaps, surprising given the social barriers participants described earlier. However, this may further demonstrate the perceptive understanding people with AS have about their difference and detachment. They may be spectators in social life but they appear to have a ringside seat. It could also be an outcome of the level of writings and discussion about ASD both in print and on the Internet as discussed earlier that people with AS draw upon to make sense of their experiences.

For some participants, getting the diagnosis of AS helped them learn more about fitting in. It seemed to give them more confidence and an acceptance for who they were. In a way, it explained their ‘strangeness’. They could incorporate AS into their identity/ies and make more sense of who they were, reframing their experiences and behaviours;

When I found out I was sad and happy. Sad that I would never be part of a world I spent a long time being on the outside of. I used to think that if I tried just a little bit harder I could belong and be accepted – it does look good sometimes, your world. To be able to laugh with different people, find things to talk about. I do not understand communication and find using voice hard.

Happy that I could stop trying so hard because I would never get there, that I could now say please stop punishing me I am trying as hard as I can – there is a reason I cannot be what you want … but in saying that there is a great pain and loneliness and black hole. (Trisha, aged 42, email interview)

This extract underlines the complicated, difficult and emotional way in which people with AS experience social life. Feeling different, for these participants, is not something to celebrate; most people wanted to feel that they belonged and were accepted for who they were. While finding out why they are different helped, it left them still separate from mainstream social life.

Participants talked about striking a balance between trying to fit in but protecting themselves from stressful situations and this led to discussions about safe spaces. Interestingly, there are some similarities here in participants' experiences and the experiences of people with agoraphobia as both find social interactions problematic. Maintaining a pretence, for example, is exhausting and, as Davidson (2003) discussed in her study of the experiences of people with agoraphobia, lead people to find solace in the home or by creating temporary or mobile senses of safety.

## 7 Safe spaces

Given the difficulties outlined above, it is not surprising that people found many social spaces difficult to negotiate and avoided everyday situations, again paralleling research involving people with phobias (Davidson, 2007b). Difficulties in communicating and understanding social cues combined with sensory sensitivities made some spaces tense and uncomfortable for participants. One man, Michael, described experiencing a “global synesthesia” in places like supermarkets and a “frightening brain overload” because of the noise and lights. Others said that they could not cope if there were more than a few people around because they could not understand what was being said or who was saying it. While some people forced themselves to go into social settings such as pubs or gigs, others talked about the spaces they felt safe in.

The safety of these spaces related to the nature of interactions they facilitated with others. Most participants found the computer and Internet offer access to a safe space in which they could interact with people with similar experiences, as the following extract illustrates;

I think computers are a big part of people on the spectrum because we know, I suppose it is the predictability thing, you just switch it on and the same screen will be there and it is not going to be a different person if you like. [um] We can relate a lot more to computers because of our wiring I think isn't it? I just think its, I don't know. Just… [5 second pause] there is a safety aspect with it as well I think and predictability is that most of us know what is going to happen. I mean nobody I know who is on the spectrum worries if the computer does something wrong, because we expect it to go wrong at some stage. And it is usually during a process that we are actually pushing it to its limit, whereas if it is with a person, I don't know whether you are going to [um] break down the conversation if you like, or not understand the conversation, or be hostile. I have got no idea of when that is going to come in and what is always a constant worry is that what if they think I am weird, what if they think I am strange, what if they don't understand, when they don't understand what will they say? (Tim, 44)

Communicating electronically removed difficulties around understanding body language and facial expressions and there was less chance of misunderstandings (see also, Davidson, 2008; Williams, 2005). Several participants discussed spending a lot of time on online forums interacting with other ‘aspies’. One young man, Tom, said how his family, several of whom were on the autism spectrum, communicated via MSN within the home as it was easier. Some people said that it was a less tiring form of communication and Trisha and Tim were both doing online courses because it was “safe”.

Another safe space people discussed was face to face support groups, rather than online forums. Some participants experienced understanding and acceptance among other people with AS as they were released from normative social expectations and some also found that their awareness of, and understanding about, autism also increased by doing so. These support groups were social events and they often met in pubs. One group, for example, met in an ‘aspie-friendly’ pub;

Oh we chose the pub purposely because it is an, it could have been made for Aspergers this pub. There is no music. No juke box. No football. No rowdy crowds. (Peter, aged 62)

The use of face to face support groups was not an indication that participants were isolating themselves as part of a wider disability/Asperger community. Some participants, for example, found interacting with other people on the spectrum helpful in the period immediately after diagnosis, but felt that the need for such interaction had tailed off. As Robert said, after the revelation that there were people similar to him, he had realised that because they had the same diagnosis did not mean they would get along together.

Domestic space offers protection from other people's presence, judgement and disorderliness and allows the self to re-establish its boundaries and coherence (Segrott and Doel, 2004) and home was also highlighted by some participants as a safe space. Homes were not always a safe space however, as safety could be compromised by living with someone. We saw above how Robert had made the decision to live alone because he found the idea of sharing a home uncomfortable and how some family members preferred to interact via MSN rather than engage in face to face communication. One woman discussed how she and her partner, who was bipolar, adapted and adjusted their home to accommodate them both;

We have our own rooms quite understandably. I have my room, he has his room which we totally need. You know, it is like otherwise we would go insane. Yes, but I mean there is things like light as well. I can't bear bright lights. You know, I have a real sensitivity to light, like electric light and I just cannot bear being under bright lights, so all the lights are really dim, but he hates being in dim light so, oh there is just this crazy situation of like trying to work out who goes where, where the light is. We have to have uplighters in the main room, so that bright light isn't shining right on me, and it has got like a dim switch so you can sort of change the lighting system. Yes. It is a bit crazy in our house really. (Caroline, aged 25)

This extract demonstrates how people create safe micro-spaces within existing places, such as the larger environment of the home. Another participant, Trisha, disliked anyone other than her children being in her home. She described how one person visited her each month and she could not wait for her to leave because she felt so ‘unsafe’ when she was there.

Participants enjoyed being outside, which again suggests similarities with people with agoraphobia. Agoraphobia, as Davidson (2000a, b) suggests, is a fear of social space rather than being outside and several participants described how much they enjoyed gardening, cycling and being out in the fresh air. Relationships with animals were also identified as significant to many participants who talked about the importance of their relationships with dogs. One woman, Jenny, for example, said she did not have a social life “like other people” and spent all her time looking after her dogs or working at a dog rescue centre. Trisha described how;

My allotment is good – peaceful and beautiful with frogs, toads, fox, – I have a grassy area which I mow and beds of veg that I share with the wildlife as it is their home I am cultivating for me – I have big trees that were there before I got there and it screens me from the other allotments so it could be anywhere. The children from school come and visit in the summer to see the pond and guess the veg and even the most disruptive child is calm and understands that they are a guest of the wildlife and so am I. (Trisha, aged 42, email interview)

As work on obsessive compulsive disorder (OCD) and agoraphobia has found (Davidson, 2000a, b; Segrott and Doel, 2004), participants were unusually sensitive to the presence and spatial practices of others in a way that most people are not. And, as the above extract suggests, it is not a generic ‘others’ because the participant found interactions with children unproblematic – they accepted her for who she was – and allowed them to enter her safe space.

## 8 An uncommon sense attitude

This analysis gives insights into both the ‘aspie’ experience and the maintenance of social order in mainstream society. Interactionism and ethnomethodology offer concepts to help explain how ‘aspies’ move through social life with concern about the contingencies, risks and hazards that exist at a micro- and macro-level. This is in contrast to neuro-typical people who draw on a common sense attitude which reduces these concerns to the level of largely taken for granted and unremarkable.

The experiences of people with AS therefore open a window into aspects of social life that are generally taken for granted (Davidson, 2000a). This analysis demonstrates the efficacy of these sociological insights and illustrates how interactionism and ethnomethodology provide tools to theorize about the position of people with AS. Both approaches emphasize the significance of socialisation and fitting in; of developing Schutz's common sense attitude or Mead's generalized other. However the key point is the significance of fitting into a very narrowly constructed version of ‘normality’. Goffman's work is key to understanding the negative consequences for those people who do not fit in and are alienated from interaction. As Goffman (1967: 115) suggests; “those who break the rules of interaction commit their crimes in jail” emphasizing the significance of following social norms and interactional rules and maintaining a working consensus for many members of society.

Using these sociological concepts we suggest that people with AS develop a different symbolic capacity, a different way of making sense of the social world which is, in some ways, in opposition to mainstream social life. People with AS can be seen to have a different logic (perhaps an ‘uncommon sense’) towards understanding the social that leads them to try to find ways of imposing orderliness and systems for managing their social world. Their systems are, however, often at odds with mainstream society. We saw how participants could learn ways of fitting in, but these remained superficial and were not, or were only partially, internalised. Instead of living their lives paying little regard to the micro aspects of everyday interactions by drawing on a stock of common sense understandings, participants had to consciously draw upon learnt behaviours and strategies to try to fit into mainstream life. This was harder when they had a break from a particular context or entered a new context.

Without the symbolic capacity to improvise within this narrowly defined symbolic system of the ‘normal’ world – which most people learn and internalise albeit to varying degrees – participants constantly experienced interactional difficulties and were perceived to be ‘faulty interactants’. They therefore lost the right to the principle of ‘civil inattention’ (Goffman, 1963) and came to expect negative and demoralizing responses from others present. Their daily lives involved a conscious focus on negotiating alien and bewildering encounters. Perhaps the barrier so many participants described (and that is also articulated in autobiographical and non-clinical literature) is related to an inability to become *spontaneously* involved in encounters. For Goffman, an inability to derive a firm sense of reality from spontaneous involvement in everyday encounters can lead to experiences of anomie and detachment, experiences clearly articulated by the participants with comments such as ‘it all seems to be happening over there’ or ‘everyone has got it, but me’.

The additional layer of complexity here is the level of awareness participants had about mainstream life. They could see it very well but could not access it fully, a position which was even more isolating, painful and distressing. They were, effectively, at the centre of an ontological crisis on a daily basis and their experiences suggest an intense ‘autistic emotion’ arising from this awareness and the accompanying fragile, precarious position they occupied in many social settings. The findings support recent psychological research into Asperger syndrome, empathy and theory of mind (see, for example, Golan and Baron-Cohen, 2006; Rogers et al., 2007) in that participants experienced emotions but were less able to understand the emotions of others without some guidance. There is an emotional detachment from other people but this does not mean that people with AS are emotionless, a point articulated by John with his description of himself as a terminator with feelings. This is an area that warrants further research, particularly given the high levels of depression and mental health issues linked to people with AS (Tantam, 2000).

There were spaces which participants considered safe, where they could interact comfortably and not be perceived as ‘faulty interactants’. The most important of these spaces was the Internet where participants could interact without many of the nuanced hazards of face to face interaction. The different symbolic capacity was less relevant in the interactional world of the Internet and there is some irony here that historically the Internet has developed in parallel to the growing recognition of Asperger syndrome.

## 9 Conclusion

Using a sociological lens within this analysis has allowed an engagement with the experiences and behaviours of participants in terms of difference rather than deficit. People with AS develop a different symbolic capacity but are very aware of ‘life over there’. The perception and insight ‘aspies’ have of mainstream life, however, is not reciprocated by neuro-typical people. While most participants were relieved to receive the diagnosis of AS – because it helped them to make sense of their experiences and they could incorporate it into their identities – they did not demonstrate a desire to isolate themselves as part of a wider Asperger community. The overriding theme was a desire to fit into mainstream society and ‘get’ its tacit rules. Given this desire and the efforts participants described to try to achieve this, future research might explore or question the moral obligation of the rest of society to facilitate and support the inclusion of people with AS in mainstream life. As we noted earlier, Goffman (1967: 116) described the mutual obligation members of society have to maintain a working consensus and facilitate the involvement of each other as “the spark that lights up the world”. That the limitations to this ideal, in practice, remain significant is, or should be, of consequence to all members of society.

## Footnotes

1 The term ‘aspie’ is a shortened version of Asperger used by many people with Asperger syndrome; see, for example, www.wrongplanet.net.

2 The methods that members of society use to make sense of, and interpret, the social world (Schutz, 1962).

3 An alternative interpretation is to view civility as a set of practices of exclusion, as in research that demonstrates how ‘others’ are kept at a distance (Bauman, 2000; Davis et al., 2004; Phillips and Smith, 2003; Sibley, 1995).

4 The theory of mind is commonly characterised as the ability to understand other people as intentional agents, to recognize or interpret their thoughts and feelings (see, for example, Frith and Happe, 2005).

5 Symbolic capacity is the ability people have to transcend their immediate surroundings and recall or envisage past and future situations (Mead, 1934).

6 The ‘natural attitude’ has two forms, the common sense attitude and the scientific attitude. Both take the existence of an external world for granted (Schutz, 1962).

7 Participants can choose the format in which they would like their interview to be included on the website. Some participants who prefer anonymity chose text only versions and should they so wish, all the identifying details and information would also be changed.

8 John is referring to the film character – the Terminator – an emotionless cyborg.

9 Within the body of autobiographical work by people with AS, Donna Williams has written an account of her ‘autistic marriage’ (Williams, 1998) offering insights into alternative ways of being and living.
